# Vision-Based Robot Navigation through Combining Unsupervised Learning and Hierarchical Reinforcement Learning

**DOI:** 10.3390/s19071576

**Published:** 2019-04-01

**Authors:** Xiaomao Zhou, Tao Bai, Yanbin Gao, Yuntao Han

**Affiliations:** College of Automation, Harbin Engineering University, Harbin 150001, China; xiaomaozhou26@gmail.com (X.Z.); gaoyanbin@hrbeu.edu.cn (Y.G.); hanyuntao@hrbeu.edu.cn (Y.H.)

**Keywords:** vision, place cell, head direction cell, unsupervised learning, hierarchical reinforcement learning, goal-directed navigation

## Abstract

Extensive studies have shown that many animals’ capability of forming spatial representations for self-localization, path planning, and navigation relies on the functionalities of place and head-direction (HD) cells in the hippocampus. Although there are numerous hippocampal modeling approaches, only a few span the wide functionalities ranging from processing raw sensory signals to planning and action generation. This paper presents a vision-based navigation system that involves generating place and HD cells through learning from visual images, building topological maps based on learned cell representations and performing navigation using hierarchical reinforcement learning. First, place and HD cells are trained from sequences of visual stimuli in an unsupervised learning fashion. A modified Slow Feature Analysis (SFA) algorithm is proposed to learn different cell types in an intentional way by restricting their learning to separate phases of the spatial exploration. Then, to extract the encoded metric information from these unsupervised learning representations, a self-organized learning algorithm is adopted to learn over the emerged cell activities and to generate topological maps that reveal the topology of the environment and information about a robot’s head direction, respectively. This enables the robot to perform self-localization and orientation detection based on the generated maps. Finally, goal-directed navigation is performed using reinforcement learning in continuous state spaces which are represented by the population activities of place cells. In particular, considering that the topological map provides a natural hierarchical representation of the environment, hierarchical reinforcement learning (HRL) is used to exploit this hierarchy to accelerate learning. The HRL works on different spatial scales, where a high-level policy learns to select subgoals and a low-level policy learns over primitive actions to specialize on the selected subgoals. Experimental results demonstrate that our system is able to navigate a robot to the desired position effectively, and the HRL shows a much better learning performance than the standard RL in solving our navigation tasks.

## 1. Introduction

The ability to perform self-localization and navigating to desired target locations is of crucial importance for both animals and autonomous robots. During interacting with environments, many animals demonstrate an inborn capability of developing spatial representations of the ambient world and are able to extract behaviorally relevant information from perceived environmental information. Early studies [1,2] linked these abilities with the formation of the “cognitive map”, which refers to an animal’s internal representation of the environment. However, for a long time, it we did not know how and where spatial cognition is formed in an animal’s body.

Recent advances in neuroscience have revealed that the formation of cognition is neurally instantiated by animals’ hippocampus and related structures [3,4], where several types of neuronal populations are found to be able to develop space-related firing properties. Among these neuron types, place cells [5] and head-direction (HD) cells [6] develop activity profiles that are closely related to an animal’s current spatial location and gaze direction, respectively. Place cells are primarily located in the CA1-CA3 regions of the hippocampus [5]. Experimental recordings on rats show that place cells only fire when a rat reaches a specific location in an environment, and their firing activities in open fields tend to be invariant to a rat’s head direction [7]. Different place cells are active in different locations of the environment. They are regarded as the neural representations of an animal’s spatial positions and encode an animal’s position with their location-selective firing characteristics. HD cells are found in many brain areas including the hippocampus and several cortical and subcortical areas [8,9]. These cells tend to significantly increase their firing rates when an animal’s orientation meets a specific direction and different cells have different preferred directions to fire. Such firing activities are independent of the location [10] and the population of these activities underpins an animal’s sense of direction. Considering their spatial awareness, these specialized navigation neurons including later found grid cells [11], border cells [12], interact with each other and collectively form a dynamic neural circuit for animals to perform self-localization, planning, and navigation.

In terms of modeling place and HD cells, there are many approaches [13,14,15] that are able to explain the formation of hippocampal representations, among which some intended to solve spatial learning for robot navigation [16,17]. Generally, these models could capture the spatially selective firing pattern of one or several cell types on different levels of abstraction, using different sensory inputs and formation mechanisms [13,15,16]. From the perspective of neural computation, the slowness principle [12,18] recently has been argued as a fundamental principle for hippocampal learning. Based on this principle, a hierarchical Slow Feature Analysis (SFA) network is able to explain the self-organization of certain hippocampal cell types, such as place and HD cells, through learning from visual stimuli in an unsupervised fashion [19,20]. In this work, we use the SFA algorithm to generate place and HD cells from vision, whose ensemble activities represent the positional and directional information, respectively.

Numerous experiments have demonstrated the essential role of hippocampus cells in solving navigational tasks including self-localization [21], orientation detection [22], route planning [23] and goal-directed navigation [24], etc. However, it is still an open question of what are the exact functional roles of these location- and direction-related cells in guiding navigational behaviors. A navigation task can be simply concluded as the process of moving from a starting location to a designated goal location, where the whole navigation process is driven by the goal. To solve such a task, Reinforcement Learning (RL) [25] provides a suitable framework by learning navigational polices during interacting with the environment. However, despite great successes in many applications [26,27,28], standard RL methods show low learning efficiency in environments with sparse and delayed reward and do not scale well for larger, more complex tasks. To solve this, hierarchical reinforcement learning (HRL) [29] provides a promising direction by breaking down complex tasks into sub-tasks through a hierarchy of abstractions. In a typical HRL paradigm, high-level policies learn to select from a series of subgoals which might only consist of several abstract, high-level observations and actions (macro-actions, options), and low-level policies learn over primitive actions to accomplish the selected subgoal. This greatly reduces each level’s search space and facilitates temporal credit assignment. Besides, the low-level policies can be re-used in tasks that consist of the same subgoals. The key problem of HRL is how to recognize the hierarchy in a learning system. While most studies [30] rely on human-designed hierarchical structures and learn policies within hardwired structures, a more plausible solution is to automatically discover these subgoals during interacting with the environment.

This work is the following work of our previous work [31,32] on realizing robot navigation based on learned place and HD cells. In our previous work [31], we extended the standard SFA algorithm to simultaneously learn separate cell types from visual images, where different cell types can be produced in an intentional way by restricting the SFA learning to different movement phases during the spatial exploration. Based on the learned cell representations which implicitly encode spatial information, we could enable a robot to perform self-localization and orientation detection. Specifically, we used a self-organized learning algorithm to learn the statistical distribution of the place cell activities that cover the traversable areas in the environment. This generates a topological map for a robot to perform self-localization. Similarly, the robot’s orientation can be denoted by a second topological map that is learned from the HD cell activities.

In this work, we move a step forward and aim to realize robot navigation based on the learned representations. To this end, we adopt RL to map the cell activities into actions that support desired navigations. In the RL paradigm, the state of the navigation system is represented by the ensemble activity of a population of place cells. During learning, the relationships between the place cell activities and behavioral actions are gradually found, where the state-to-action mapping function is built to support corresponding navigational behaviors. Considering the challenge of learning in large and complex environments, we scale up RL to HRL for better learning performance and efficiency. The topological map, which is built through clustering the place cell activities, represents the environment on a high-abstract level and provides a natural hierarchy for facilitating HRL. Particularly, the map is built in a self-organized fashion during exploring the environment without any human efforts. In our work, the HRL consists of two layers of policies that work on different spatial scales. The high-level policy works within the topological map to select which subgoal to complete, where the subgoal refers to the node in the topological map. The low-level policy works in the space of place cell activities and chooses primitive actions to achieve the selected subgoal. Combining these together, we present a navigation system that is able to perform goal-directed navigation based on learning representations from vision, where intermediate processes involve modeling place and HD cells and building topological maps for self-localization and orientation detection. Note that the focus of this work is not the hippocampal modeling, instead, we want to build a pragmatic navigation system based on the learning representations from the SFA model. Particularly, we highlight our contributions in this paper as follows:

- We present a modified SFA learning model to produce place and HD cells by learning directly from visual images in an unsupervised fashion. The proposed approach is able to learn different cell types simultaneously by restricting the learning to separate phases of spatial explorations.
- We propose a self-organized method to extract spatial information from unsupervised learning representations, which generates topological maps and enables a robot to perform self-localization and orientation detection.
- We perform goal-directed navigations by applying HRL to map the population activities of place cells into actions that support corresponding behaviors. The hierarchy is provided by a topological map which is built in an unsupervised fashion during interacting with the environment.
- Simulation experiments including comparisons with the standard RL method demonstrate the validity and effectiveness of the proposed navigation system in performing goal-directed navigations based only on vision.

For the following parts of this paper: Section 2 gives a brief review of the existing modeling approaches and navigation systems incorporating hippocampal cells, together with HRL techniques. Section 3 introduces the adopted algorithms in this work, including the SFA algorithm, Growing When Required (GWR) network, and RL. Section 4 first presents our modified learning approach that enables the separate learning of place and HD cells and then elaborates the process of building topological maps, and finally explains the implementation details of using HRL to solve navigation tasks. Section 5 presents the experimental results and compares our system with several different approaches, which demonstrates the efficiency of the proposed system in solving goal-directed navigation tasks. Finally, the paper ends with a general discussion of our system in Section 6 and a conclusion in Section 7.

## 2. Related Work

### 2.1. Hippocampal Modeling and Related Navigation Systems

Ever since their initial discovery in rodents’ hippocampus, place cells, which have demonstrated their prominent roles in spatial representations [11] and episodic memory [33], have attracted substantial attention from different research communities. For the hippocampal modeling, various approaches have been proposed in an attempt to explain these spatially sensitive firing activities, such as Gaussian function [16], neural plasticity [13,14], self-organizing map [15], etc. More biologically motivated models suggested that place cells are originated from grid cells and generated place cell responses from a subset of grid cell inputs [34,35]. However, although many models were able to systematically develop the location- or direction-related firing patterns, few cared about the computations happening in the hippocampus and there was little discussion over the computational principle underlying the formation of these activities. Throughout the literature, only the SFA model [20] tried to explain this problem from a computational point of view and was able to produce signals featuring firing activities found in hippocampal cells by learning from raw sensory inputs. The underlying learning mechanism is important since it reveals the detailed implementation of how the brain utilizes the sensory information to extract behaviorally relevant features and to direct the behavioral choices. This work uses the SFA algorithm to generate place and HD cells from visual inputs only.

There also exist some neurobiologically inspired navigation systems that intended to accomplish spatial awareness by incorporating one or several types of navigation-related cells. For example, RATSLAM model [36] proposed to represent spatial information in synthesized pose cells, in which the pose cells are represented in a 3-D continuous attractor network (CAN) by integrating internal sensing and external vision perception. The pose cells in their work can be regarded as a combination of place cells and HD cells. Tejera et al. [37] developed a neural grid cell model to represent ’neural odometry’ which is integrated to place cells in the representation of different locations in the environment. For navigation, Giovannangeli et al. [38] presented a hippocampal model performing transitions and plannings based on the gradient diffused from the goal on top of the learned place cell representations, which enables to reach a goal from a random position. Some works [13,39] employed a reinforcement learning paradigm to accomplish place cells’ functional role in goal-oriented navigation tasks, in which the states are represented by locations encoded in the population activity of place cells and the state-action value is learned online with respect to a specific goal during searching phases. Besides, to demonstrate the predictive ability of the hippocampal cells, Erdem et al. [40] presented a goal-directed navigation model that allows for finding direction to remembered goal locations based on the interconnections of HD cells, place cells, and grid cells. However, they utilized many elaborate mechanisms in order to explain the emergence of different neuron types according to the movement, rather than using the emerged firing activity to direct behavioral choices.

Additionally, in our previous work, we have presented two different models that use the learned place and HD cells to perform navigations. In the first work [32], based on the learned place and HD cells, we presented a neural model that enables to build topological maps and perform navigation in a self-organized manner, where a behavior-imitation mechanism is used to learn actions that support state transitions and the robot is able to reach the target in a reward-ascending way. However, anchoring action memories into the map requires lots of human efforts. In the second work [41], we proposed a hybrid planning strategy that includes performing look-ahead planning based on a world model on the global scale and object-driven reaching based on visual recognition on the local scale. This enables a robot to navigate to a distant target with finer accuracy. However, the final performance of this model relies heavily on the accuracy of the trained world model. In this work, we aim to build a navigation system that is free of this requirement and is able to automatically learn navigational behaviors during interacting with the environment.

### 2.2. Hierarchical Reinforcement Learning

The basic idea of HRL is to break down a complex RL task into several sub-tasks which are easier to solve through a hierarchy of abstractions. Compared with the standard RL which requires an agent to make decisions on each primitive action at every granular state, HRL could abstract a series of micro-decisions into a macro-decision and focuses on a small set of important decisions, thus leading to a much faster learning speed. HRL has shown its superiority in solving many tasks involving long-range delayed rewards. For example, Kulkarni et al. [42] presented a hierarchical-DQN framework that integrates the temporal abstraction and an intrinsic motivation mechanism to be able to play the classic Atari game ‘Montezuma’s Revenge’ which is a big challenge for standard RL approaches. Tang et al. [43] used HRL to learn the dialogue policy for task-completion dialogue agents, where subgoals in the complex goal-directed task are automatically learned in an unsupervised fashion. Peng et al. [44] showed that the use of subgoals mitigates the reward sparsity and leads to more effective exploration for learning.

Within the HRL paradigm, Sutton et al. [45] proposed the options framework which provides a natural way for hierarchical policy learning, where an option is a generalization of primitive actions to include temporally extended courses of action. However, it remains a troublesome problem of how to find the options or how to define the hierarchy in a learning task, which is the main research topic in relevant research areas. In terms of hierarchical formulations, many previous works assume that the hierarchical structure is given by a designer, which requires the domain knowledge and many human’s efforts. It is desirable for a learning system to learn the hierarchy itself. For this aim, methods based on varying reward functions [46] or by composing existing options [47] have been proposed to learn options in real-time. There are also lots of work on option discovery in the tabular value function setting. For practical implementations, Goel [48] made use of the structure present in a particular environment to discover subgoals by studying the dynamics along the predecessor count curve, where the discovered subgoals usually refer to the junctions between different rooms. Csimcsek et al. [49] used the betweenness centrality measures in a graphical representation of the environment to identify subtasks. Menache et al. [50] adopted the clustering method to separate the strongly connected components of a Markov Decision Processes (MDP) into different clusters that are regarded as subgoals. Similarly, Lakshminarayanan et al. [51] addressed the automated option discovery in HRL using spatio-temporal clustering. In this work, we identify the subgoals in our navigation task within a topological map which is built in a self-organized way during exploring the environment, where the self-organized learning can also be regarded as a clustering method.

## 3. Materials and Models

### 3.1. Slowness Principle

Slowness principle claims that more meaningful information within an input data stream is capable of keeping its consistency for a relatively long period of time, while less meaningful information always varies quickly. Considering an image sequence depicting a moving object, the object’s moving trajectory always changs in a continuous way and can be easily captured by an observer, while for the intensity value change of a single pixel, it changes too fast and contains no interpretable information. Based on this principle, high-abstract features within the sensory input can be extracted in an unsupervised fashion by considering their varying speed.

### 3.2. Slow Feature Analysis

SFA is an unsupervised learning algorithm implementing the slowness principle. For consecutive raw sensory, SFA is able to capture the slowly varying signals and leaves out quickly changing ones. In most cases, these slowly varying features always contain the most descriptive statistical regularities. For example, consider a mobile agent exploring an environment with its visual system, the change of its visual information is always related to the continuously varying positions and orientations, where the emerging slow features compactly encode this information. Sufficient results from both theoretical analyses and experimental tests have demonstrated that SFA is able to model certain types of hippocampal cells by directly learning from raw visual inputs [18]. Combined with a sparse coding mechanism, a localized firing activity similar to that found in the hippocampus can be organized in a clear pattern [19].

Mathematically, the SFA learning problem can be described as follows: Given an *I*-dimensional input signal $x\left(t\right)=[x_{1}\left(t\right),x_{2}\left(t\right),\ldots ,x_{I}\left(t\right)]$, find a set of *J* real-valued input-output functions $g\left(t\right)=[g_{1}\left(t\right),g_{2}\left(t\right),\ldots ,g_{J}\left(t\right)]$ such that the output signal $y\left(t\right)={\left[y_{1}\left(t\right),y_{2}\left(t\right),\ldots ,y_{J}\left(t\right)\right]}^{T}$ with $y_{j}\left(t\right)=g_{j}\left(x\left(t\right)\right)$ satisfies the criteria:

(1) $$\Delta \left(y\right)={\langle {\dot{y}}_{j}^{2}\rangle }_{t}\;is\;minimal$$

under three constraints:

$${\langle y_{j}\rangle }_{t}=0\;zero\;mean$$

$${\langle y_{j}^{2}\rangle }_{t}=1\;unit\;covariance$$

$$\forall j^{\prime }<j:{\langle y_{j^{\prime }}y_{j}\rangle }_{t}=0\;decorrelation$$

where 〈·〉 and $\dot{y}$ indicate the temporal averaging and the time derivative of *y*, respectively. Equation (1) represents the primary objective of the optimization problem. The first two constraints are used to guarantee the output signals with meaningful information, rather than a trivial constant value. Decorrelation is used to avoid uninteresting solutions where all the output signals encode the same information.

### 3.3. Growing When Required Network

The self-organizing network Growing When Required (GWR) [52] is a kind of neural networks inspired by biological self-organization and is able to learn the important topological relations in a given set of vectors in an unsupervised way. Different from previous approaches like the Self-Organising Map (SOM) [53] and the Neural Gas (NG) [54], GWR does not need to define the size of the network beforehand and exerts a dynamic growing mechanism to enable the network to grow automatically when the current nodes cannot represent the inputs accurately. This enables it to respond quickly to changes in the input distribution by dynamically learning, adding or deleting nodes and connections.

During learning, a GWR network starts with two random nodes $n_{1}$ and $n_{2}$ representing the input space. For each iteration, two best matching nodes *s* and *t* are selected based on the distance to the input, where these two nodes are always connected. Whenever *s* and *t* fail to represent the current input with a certain accuracy, a new node will be inserted halfway between them. The criterion of adding new nodes is also dependent on the firing counter of the best node. Training will drive the weights of the best matching node and its neighbors towards the input and the rarely used nodes will be deleted by an aging mechanism. The algorithm will keep iterating until meeting a stop criterion, such as the desired performance has been met or the network has reached the maximum size. The learning steps of GWR are described as follows:

1. Start with two neurons $n_{1}$ and $n_{2}$ with random weights $w_{n_{1}}$ and $w_{n_{1}}$.
2. Generate an input signal ζ (place cell activity vector) according to the place cell network.
3. Find the nearest neuron *s* and second-nearest neuron t according to the distance from the input: $∥\zeta -w_{i}∥$.
4. If there is no connection between *s* and *t*, create it. Otherwise, reset the age of this connection to zero. Calculate the activity of each neuron *i*: $s_{i}=exp(-∥\zeta -w_{i}∥/2\delta^{2})$
5. If $s_{s}<$ activity threshold $a_{T}$ and firing counter $h_{s}<$ firing threshold $h_{T}$, insert a new neuron as follows:
  - Add a new neuron *r* halfway between the best matching neuron and current input: $w_{r}=\left(w_{s}+\zeta \right)/2$
  - Insert connections between *s* and *r* and *t* and *r*
  - Remove the connection between *s* and *t*
  Else, i.e., no new neuron is added, adapt the positions of the best matching neuron *s* and its neighbours *i*:
  - $\Delta w_{s}=\epsilon_{b}\cdot h_{s}\cdot \left(\zeta -w_{s}\right)$
  - $\Delta w_{i}=\epsilon_{n}\cdot h_{i}\cdot \left(\zeta -w_{i}\right)$
  where $0<\epsilon_{n}<\epsilon_{b}<1$ are learning rates and $h_{s}$ is the value of the firing counter for node *s*.
6. Age connections with an end at *s*: $age_{\left(s,i\right)}=age_{\left(s,i\right)}+1$
7. Reduce the firing counters of neuron *s* and its neighbours:
  - $h_{s}\left(t\right)=h_{0}-\frac{S(t)}{\alpha_{b}}\left(1-e^{\left(-\alpha_{b}t/\tau_{b}\right)}\right)$
  - $h_{i}\left(t\right)=h_{0}-\frac{S(t)}{\alpha_{n}}\left(1-e^{\left(-\alpha_{n}t/\tau_{n}\right)}\right)$
  where $h_{0}$ is the initial strength and *S* is the stimulus strength. $\alpha_{b}$, $\alpha_{n}$ and $\tau_{b}$, $\tau_{n}$ are learning constants.
8. Remove all connections with ages larger than $a_{max}$ and remove neurons without connections.
9. If the stopping criterion is not yet fulfilled, go to step 2.

### 3.4. Deep Reinforcement Learning

Reinforcement Learning (RL) is an important type of machine learning techniques where an agent learns in an interactive environment by trial and error using feedback from its own actions and experiences. In RL, an agent interacts with an environment over a series of time steps. At each time step *t*, the agent perceives a state $s_{t}$ and needs to select a possible action $a_{t}$ according to an action-selection policy π, where the π is the probability of selecting an action a to be performed for a given state s. Executing the selected action $a_{t}$ leads the agent to the next state $s_{t+1}$, and the agent also receives a reward *r* from the environment. During learning, the agent’s aim is to find the optimal policy $\pi^{*}$ that maximizes the expected value of reward received over time.

Given a policy π, the action-value (Q-value) of a state-action pair (s, a), which indicates the expected total discounted reward when executing actions following policy π from state s, is defined as follows:

(2) $$Q^{\pi }\left(s,a\right)=E\left[\sum_{t=0}^{\infty }\gamma^{t}r_{t}|s_{0}=s,a_{0}=a,\pi \right]$$

where the expectation is with respect to the transition distribution under policy π and $r_{t}$ is the reward for action $a=a_{t}$ under the policy π in the state $s=s_{t}$. γ is the discount rate determining future action’s influence (0<γ<1).

Based on the Bellman equation, the optimal $\pi^{*}$ corresponds to taking the best action in any state *s* where $Q^{*}\left(s_{t},a\right)=max_{\pi }E\left[R_{t}|s_{t},a,\pi \right]$ and the optimal Q-value function $Q^{*}$ can be obtained as follows:

(3) $$Q^{*}\left(s,a\right)=r_{t}+\gamma \max_{a^{\prime }}E\left[Q^{*}\left(s_{t+1},a^{\prime }\right)\right]$$

where $a^{\prime }$ represents the possible actions in the future state $s_{t+1}$.

Recently, the technique of combing deep neural network and RL, where neural networks work as function approximators, has shown promises in handling high-dimensional sensory inputs and has extended RL to a large variety of applications. This is the main principle behind (Deep Reinforcement Learning) DRL [55,56].

## 4. Proposed Approach

The proposed navigation system is based on the learning representations from vision. Specifically, it involves producing place and HD cells through learning from visual images using the modified SFA algorithm and building topological maps based on the learned cell activities. The topological map then provides a natural hierarchy for implementing HRL. We will briefly introduce them in this section.

### 4.1. Training Different Cell Types from One Exploration

For the place and HD cells learning, we use the SFA algorithm to learn directly from a robot’s visual images during exploring the environment. Considering that the SFA is a purely sensory-driven model whose learning is based on the concept of temporal stability among consecutive input stimuli, the temporal structure of the sensory data for training will be closely related to the final representations. This provides us with the possibility of varying the emerged firing patterns by manipulating the input statistics. For this aim, during modeling different cell types, a robot is driven to actively explores the environment with its visual system and its visual images during different movement phase are collected for separate training. Specifically, during forward movements, where the robot’s position continuously changes and its direction rarely changes, the emerging slow features during these phases compactly encode the robot’s moving direction and are used to model HD cells. Similarly, images from the rotational movements can be used to train the place cell network. After training, with these two networks, the response of a network to a single image which is captured at a certain position to a certain direction will approximate the place cell activity at that position or the HD cell activity to that direction.

**Figure 1:**
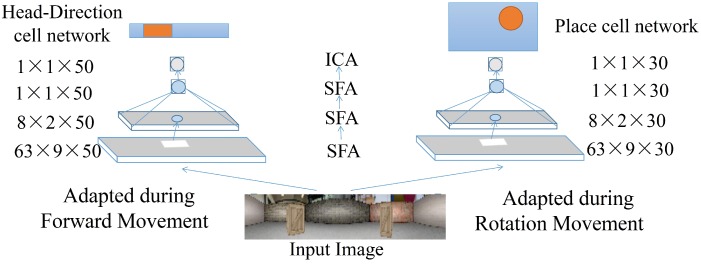
Training place cell and head-direction cell networks in different phases of the same trajectory. Layers are trained sequentially from bottom to top.

The model configurations and the training process are illustrated in Figure 1. The model consists of two parallel visual processing channels. Each channel consists of a hierarchical architecture including 3 SFA layers and 1 ICA (independent component analysis) layer. For each SFA layer, a certain number of SFA nodes are organized in a regular grid, where each node contains 30 or 50 output channels (cells) and acts on a local receptive field. The first layer has 63×9 SFA nodes working directly on the raw input images and each node extracts features based on the slowness principle from its own local-field area. Neighboring nodes cover overlapping areas, which facilitates feature detection over the whole input frame. The second layer has 8×2 SFA nodes working on the outputs of the first layer and extracting more abstract features than the first layer. The third layer has only one SFA node that integrates the outputs of all nodes from previous layers, outputting even higher abstract features. On top of the SFA layers, there exists one ICA node that performs sparse coding on the raw SFA outputs to produce a more localized representation. The outputs of the 50 units in the final SFA node of the HD cell network represent the firing activities of 50 HD cells and the 30 units in the final SFA node of the place cell network represent the firing activities of 30 place cells. We use 50 HD cells outnumbering 30 place cells in order to increase the precision of the direction estimation. For the software implementation, we use the Modular toolkit for Data processing (MDP) library [57], which provides a complete implementation of SFA and ICA. For the training data collection, we collect visual data during the forward movement period to train the HD cell network. Similarly, we collect data from the robot’s *turning movement* to train the place cell network. This mechanism is related to the assumption that learning is modulated by behavior and, more specifically, that transitional and rotational motion can be differentiated to train different types of cells. This can in principle be supported by the biological findings where place and HD cells demonstrate the ability of the behavioral modulation [8,58].

### 4.2. Topological Maps Building

The firing activities of these cells demonstrate a strong firing preference to different positions or directions, where the population activity of place and HD cells encode a certain position and direction, respectively. However, in our work, the place and HD cells are generated by the unsupervised SFA algorithm. Different from supervised learning paradigms, these cell activities have no predefined relationships to a real-world position or direction. To interpret the encoded spatial information, we adopt the GWR network to learn from the emerged cell activities. The GWR is able to capture the internal relationships of these activities and to reveal the topology of the input space. For example, during learning from place cell activities that cover the whole environment, a GWR network can generate a topological map of the explored areas, where GWR nodes represent the spatial positions and connections represent connectiveness between positions. During learning, the GWR starts with two nodes and grows incrementally during exploring the environment. In order to capture the distribution of the place cell activities, nodes and connections are created or updated dynamically and are also deleted if needed. After learning, the GWR network consists of a set N of nodes and a set C of connections that represent the relations between each connected nodes pair in N.

In our previous work [32], we were able to realize robot self-localization and orientation detection based on the PC-GWR and HD-GWR which are built by learning from the place and HD cell activities, respectively. Specifically, a robot’s position can be calculated by finding the best matching node in the PC-GWR network, where its position is represented by an ensemble activity of place cells. Similarly, the robot’s orientation can be represented by the best matching node in the HD-GWR network. In this part, we adopt the approaches in our previous work [32] to build topological maps. The map building process follows the concept of mapping high-dimensional data into low-dimensional space for practical usages, which has been studied a lot based on both supervised and unsupervised learning approaches [59,60,61].

**Figure 2:**
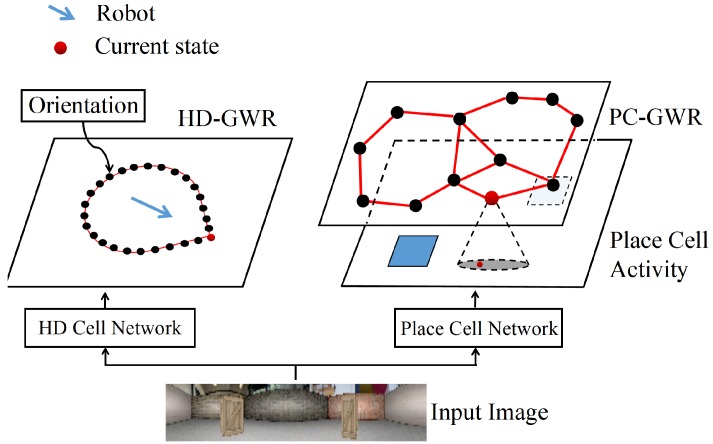
An illustration of performing orientation detection and self-localization using the built topological maps. The robot’s orientation is represented by the best matching node (shown in red) in the HD-GWR. The robot’s current location is represented by the best matching node (shown in red) in the PC-GWR.

Importantly, the topological map provides a natural hierarchy for implementing HRL, where a robot’s position in the environment can be represented on two different levels of abstraction. On the high level, it is represented by the node in the PC-GWR network. On the low level, it is represented by the ensemble activity of place cells. Each state on the high level covers a sub-area on the low level. An illustration is presented in Figure 2.

### 4.3. Hierarchical Reinforcement Learning

For the HRL implementation, we use the deep Q-Learning framework [55] to learn both the high-level and low-level policies, whose Q-functions $Q_{H}\left(s,g;\theta_{H}\right)$ and $Q_{L}\left(s,g,a;\theta_{L}\right)$ are approximated by neural networks with parameters $\theta_{H}$ and $\theta_{L}$, respectively.

- The high-level looks at the state representations (nodes) in the PC-GWR network and learns a policy $\pi_{H}$ over these nodes (subgoals) to select the subgoal for the low-level policy to complete. $Q_{H}\left(s,g;\theta_{H}\right)$ measures the maximum total discounted extrinsic reward $r^{e}$ received by choosing subgoal g in state s while following the policy $\pi_{H}$. The extrinsic reward $r^{e}$ is received from the environment and is the objective to be maximized by the entire navigation policy.
- The low-level takes in states (in the space of place cell activities) and the current subgoal (one of the PC-GWR nodes) and produces a policy $\pi_{L}$ of actions to accomplish the selected subgoal. $Q_{L}\left(s,g,a;\theta_{L}\right)$ measures the maximum total discounted intrinsic reward $r^{i}$ received to achieve a given subgoal. The low-level policy terminates either when the goal is accomplished or when the step number in the current goal reaching task reaches the maximum. The intrinsic reward $r^{i}$ is given based on whether the desired subgoal has been reached and is used to help learning how to achieve the given subgoal.

Suppose that the PC-GWR network consists of a set N of nodes $n=\{n_{0},n_{1},\ldots ,n_{N}\}$, a navigation task consisting of T transitions: $\tau =\{s_{0},a_{0},r_{0},\ldots ,s_{T}\}$, which covers a sequence of subgoals $g_{0},g_{1},g_{2},\ldots \in N$. Consider reaching the subgoal g requires M steps of transitions from t.

Following the Q-learning rules, $Q_{H}\left(s,g\right)$ can be learned by treating subgoals as temporally extended actions:

(4) $$Q_{H}^{*}\left(s,g\right)=\sum_{t^{\prime }=0}^{M}\gamma^{t+t^{\prime }}r_{t}^{e}+\gamma \max_{g^{\prime }}E\left[Q^{*}\left(s_{t+M},g^{\prime }\right)|s_{t}=s,g_{t}=g,\pi_{H}\right]$$

where the expectation is with respect to the transition distribution under policy $\pi_{H}$ and $g^{\prime }$ represents the possible subgoals in the state $s_{t_{1}}$. γ∈[0,1] is a discount factor.

**Figure 3:**
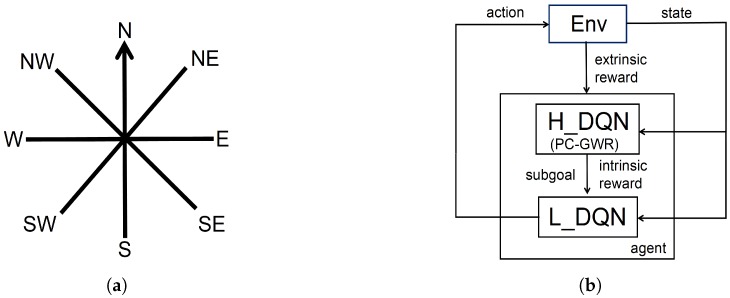
(**a**) The primitive actions used in the learning. (**b**) The HRL learning architecture.

Similarly, $Q_{L}\left(s,a,g\right)$ can be learned as follows:

(5) $$Q_{L}^{*}\left(s,g,a\right)=r^{i}+\gamma \max_{a^{\prime }}E\left[Q^{*}\left(s_{t+1},g,a^{\prime }\right)|s_{t}=s,g_{t}=g,a_{t}=a,\pi_{L}\right]$$

where $a^{\prime }$ represents the possible subgoals in the state $s_{t+1}$. In this work, the actions are defined to control the robot’s moving direction and include 8 discrete components as shown in Figure 3a.

During learning, the high-level and low-level policies use temporal difference learning at different temporal resolutions. The learning architecture is shown in Figure 3b. A high-level step corresponds to a state switch in the PC-GWR network and $Q_{H}$ learns from the state transitions $\left(s_{t},g_{t},\sum_{t=0}^{M}r_{t}^{e},s_{t+M}\right)$. A low-level step corresponds to a state transition in the space of place cell activities and $Q_{L}$ learns from state transitions $\left(s_{t},g_{t},a_{t},r_{t}^{i},s_{t+1}\right)$. A high-level time step consists of a number of M consecutive low-level steps. The combination of high-level and low-level learning enables an agent to accomplish a complex navigation task as fast as possible, where the learnings are driven by both the extrinsic and intrinsic rewards.

The objective of Q-learning is to bring the current Q-values of $Q_{H}\left(s,g\right)$ and $Q_{L}\left(s,g,a\right)$ to the target values shown in Equations (4) and (5), respectively. Recently, Van Hasselt et al [62] have proposed a Double Q-learning framework that demonstrates better performances than DQN in many tasks. Different from DQN that performs learning using only one set of parameters θ, D-DQN uses two sets of parameters θ, $\theta^{-}$ to separately determine the greedy policy and evaluate the policy value, which has shown better performance in many tasks. Based on this, $\theta_{H}$ and $\theta_{L}$ are optimized according to:

(6) $$\begin{array}{cc} \theta_{H}\leftarrow & \theta_{H}+\alpha \left(\sum_{t^{\prime }=0}^{M}\gamma^{t+t^{\prime }}r_{t}^{e}+\gamma Q_{H}\left(s_{t+M},arg\max_{g}Q\left(s_{t+M},g,\theta_{H}\right);\theta_{H}^{-}\right)-Q_{H}\left(s_{t},g;\theta_{H}\right)\right)\nabla_{\theta_{H}}Q_{H}\left(s_{t},g;\theta_{H}\right) \end{array}$$

(7) $$\begin{array}{cc} \theta_{L} & \leftarrow \theta_{L}+\alpha \left(r^{i}+\gamma Q_{L}\left(s^{\prime },g,arg\max_{a}Q\left(s,g,a;\theta_{L}\right);\theta_{L}^{-}\right)-Q_{L}\left(s,g,a;\theta_{L}\right)\right)\nabla_{\theta_{L}}Q_{L}\left(s,g,a;\theta_{L}\right) \end{array}$$

where $\theta_{H}$ and $\theta_{L}$ are the parameters for the online networks and $\theta_{H}^{-}$ and $\theta_{L}^{-}$ are parameters for the target networks. γ is the learning rate (see Algorithm 1).
**Algorithm 1** Hierarchical Reinforcement Learning 1:Initialize experience replay memories $\left\{D_{H},D_{L}\right\}$ and the exploration probability $\epsilon_{H}=1$ and $\epsilon_{L}=1$ for the high-level and low-level policies, respectively. 2:Initialize parameters $\theta_{H},\theta_{L}$ for the online networks and $\theta_{H}^{-},\theta_{L}^{-}$ for the target networks. 3:For *i* = 1, *num_episodes* do 4: Initialize the navigation and get the start state s. 5: While s is not teriminal do 6:  R←0 7:  $s_{0}\leftarrow s$ 8:  With probability $\epsilon_{H}$ select a random subgoal g from the PC-GWR nodes 9:  Otherwise select $g=\max_{g}Q_{H}\left(s,g;\theta_{H}\right)$10:  while not (s is terminal or goal g is reached) do11:   With probability $\epsilon_{L}$ select a random action a12:   Otherwise select $a=\max_{a}Q_{L}\left(s,g,a;\theta_{L}\right)$13:   Execute a and obtain the next state $s^{\prime }$ and extrinsic reward $r^{e}$ from the environment14:   Obtain the intrinsic reward $r^{i}$15:   Store transition $\left(\left\{s,g\right\},a,r^{i},\left\{s^{\prime },g\right\}\right)$ in $D_{L}$16:   Sample random mini-batches from $D_{L}$17:   Update $\theta_{L}$ according to Equation (7)18:   $R\leftarrow R+r_{e}$19:   $s\leftarrow s^{\prime }$20:  end while21:  Store transition $\left(s_{0},g,R,s^{\prime }\right)$ in $D_{L}$22:  Sample random mini-batches from $D_{H}$23:  Update $\theta_{H}$ according to Equation (6)24:  Every C steps copy $\theta_{H}$ to $\theta_{H}^{-}$ and $\theta_{L}$ to $\theta_{L}^{-}$25: end while26: Anneal $\epsilon_{H}$ and $\epsilon_{L}$ adaptively27:end for

## 5. Results

### 5.1. Experiment Setup

**Figure 4:**
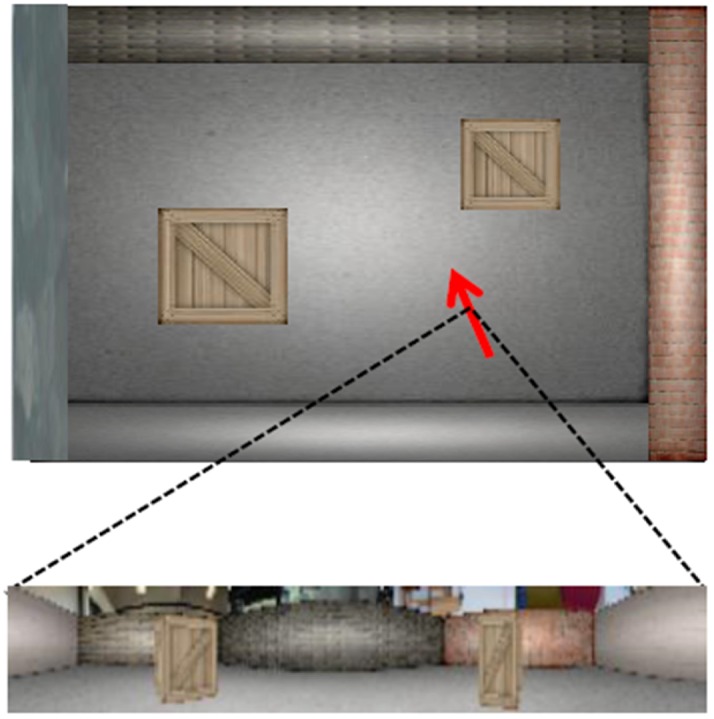
The top view of the RatLab environment rendered in this work. Below is an image (320×40) seen by the robot at the current position (indicated by the red arrow whose direction represents the robot orientation).

To test our proposed navigation system, we use a simulated robot moving in a virtual-reality environment, RatLab, which is also used to generate images for training place- and HD cell networks [63]. RatLab is a specialized simulator targeting at establishing place and HD cells based on the SFA algorithm. It is designed to simulate a virtual rat foraging in a home-like environment and allows to modify the rat’s movement pattern over the course of exploration. In addition, according to application purposes, users can modify the environment by defining the simulator shape, changing the environmental textures, and adding customized obstacles, etc. Due to its convenience and flexibility, it can also be used to simulate experiments in robotic scenarios. In this work, we test each component of our navigation system in RatLab. An overview of the simulated rectangle environment is shown in Figure 4, where an image captured by the virtual robot from a given position with a random head direction in the environment is also presented in the lower part. The simulated robot has a field of view (FoV) of 320 degrees in order to simulate a rat’s wide FoV [64].

### 5.2. Generating Place and HD Cells from One Exploration

**Figure 5:**
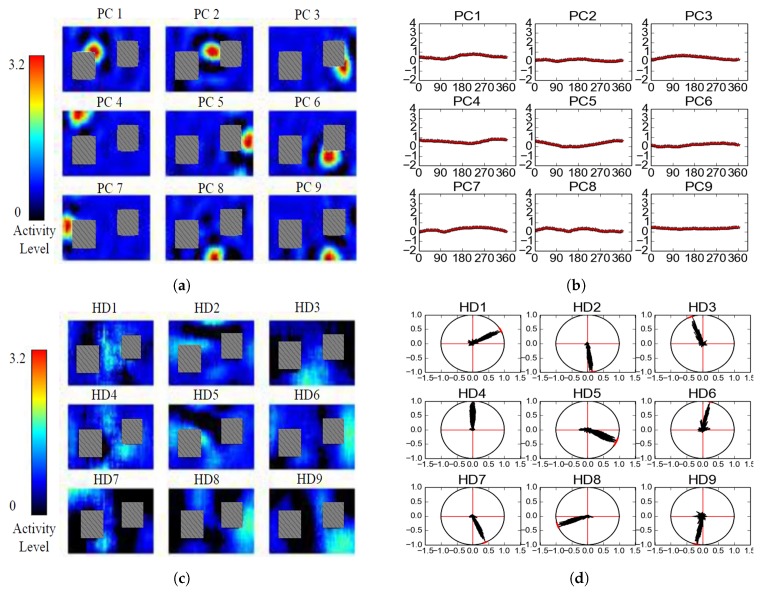
The firing activities of the trained Place and HD cells (9 randomly chosen place cells are shown in (**a**,**b**), and 9 head-direction cells are shown in (**c**,**d**)).

In this work, considering the size of the simulation environment, we trained 30 place cells, whose overlapping firing fields cover the whole environment densely, and 50 HD cells whose ensemble activity encodes the spatial position and direction, respectively. In particular, we model more HD cells in order to represent the direction with a higher precision since the direction is more important during movement. For the training, we collected 8000 images from the *turning movement* to train the place cell network and 10,000 images from the *forward movement* to train the HD cell network. Parts of the training results can be seen in Figure 5. The learned place cells only fire in a certain position in the environment (Figure 5a) and they have little directional tuning, which means their activities are invariant to the direction (Figure 5b). HD cells show little position preference (Figure 5c), but they will be significantly active when it comes to their preferred direction (Figure 5d).

In particular, we adopt the concept of entropy [65] to assess the distinguishable firing properties obtained by these two different cell types. For a set of distributions using these activities as their probability values. Since place cells have similar probabilities for different directions, their activities closely approximate a uniform random distribution and thus have a large entropy of direction $H_{dir}$. In contrast, head-direction activities are more peaked since they have large probability values for a certain direction, thus having a smaller entropy $H_{dir}$. The entropies are calculated by:

(8) $$\begin{array}{cc} H_{dir}=-\sum_{\theta }^{dir}a_{\theta }\ln\left(a_{\theta }\right); & H_{pos}=-\sum_{i}^{pos}a_{i}\ln\left(a_{i}\right) \end{array}$$

where $a_{\theta }$ represents the normalized cell activity at direction θ averaged over positions, while $a_{i}$ represents the normalized cell activity at position *i* averaged over directions.

**Figure 6:**
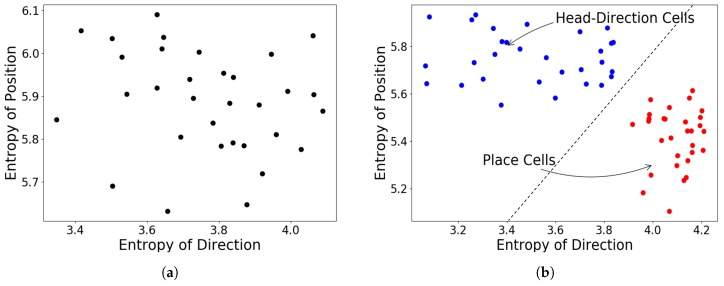
Entropy analysis: (**a**) Training results from standard SFA, without knowledge to distinguish different cell types. (**b**) With our proposed training method, different cell types form two separate clusters.

Figure 6b shows that the obtained cell types demonstrate clearly different properties. For comparison, we also present the training result of the existing model [66] in Figure 6a, where a continuum is between place and head-direction cells.

### 5.3. Building Topological Maps Based on GWR

**Figure 7:**
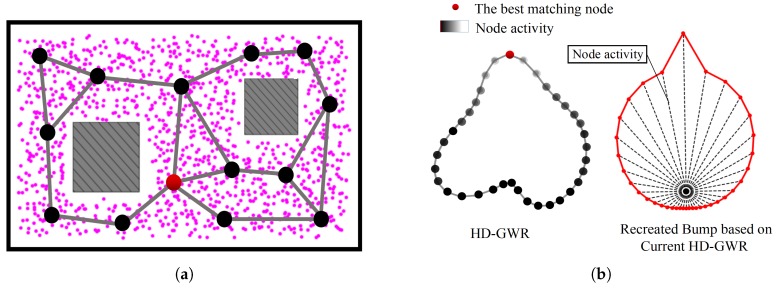
Topological maps (visualized by MDS) building based on GWR. (**a**) The learned PC-GWR represents the topology of the explored area and the robot’s current position is represented by the best matching node (the red node). The pink dots represent the positions where place cell activities are sampled during the PC-GWR learning. (**b**) Left: the built HD-GWR through learning from HD cell activities and the robot’s current orientation is represented by the best matching node (the red node). Right: a bump created based on the current HD-GWR to show the firing activity of each node.

Based on the learned place and HD cells, two topological maps of PC-GWR and HD-GWR are generated, which are shown in Figure 7. Considering that the inputs to the GWR networks are high-dimensional activities, for visualization, the multidimensional scaling (MDS) algorithm [67] is used to project the high-dimensional learning results into the 2D space. For more details on this part, please refer to our previous work [32].

At the beginning of the map building, the PC-GWR is initialized with two nodes. During driving the robot to explore the environment where each position is represented by an ensemble activity of the modeled 30 place cells, the activities along the moving trajectories are continually fed to the PC-GWR. The PC-GWR grows incrementally in order to represent the distribution of the input place cell activities. This process continues until the exploration ends. The resultant PC-GWR network gives rise to a topological map of the explored area. As shown in Figure 7a, the PC-GWR represents the environment at a coarse level and each node automatically segments the positions in the space of place cell activities into abstractions (clusters). The robot’s real-time location can be represented by the best matching node in the PC-GWR. Since our aim is to use the topological map to perform navigation based on HRL, rather than pure self-localization, we use a PC-GWR with only 14 nodes.

The HD-GWR is built through learning from HD cell activities covering a robot’s entire angle range of 360°. As shown in Figure 7b, the learning gives birth to in a ring-shape HD-GWR network which accords with the circular orientation space. Although the HD cell activities encode no explicit directions due to the unsupervised SFA learning, the robot’s current orientation can be represented by the best matching node among the HD-GWR nodes. The HD-GWR has a size of 36 nodes in an attempt to represent the direction in 10° increments.

### 5.4. Performing Navigation Based on HRL

In this section, we performed the goal-directed navigation by implementing HRL based on the learned representations described above. Additionally, we compare the performance of the proposed approach with several baselines: the standard D-DQN and two variations of our proposed approach.

#### 5.4.1. Task Setup and Training Process

**Figure 8:**
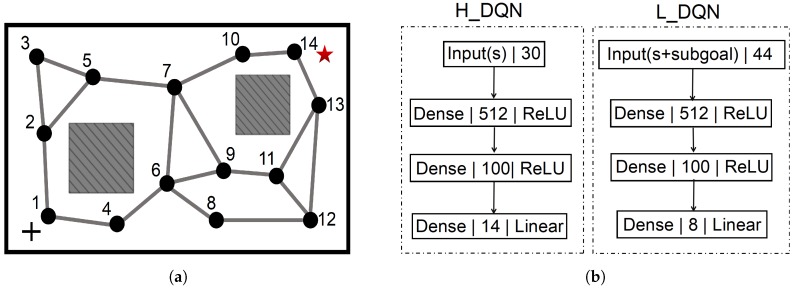
(**a**) The navigation task in our work. (**b**) The network structures of the H_DQN and L_DQN. Each layer is represented by its layer type, dimension, and activation mode.

The training and navigation are implemented in the RatLab (shown in Figure 4) whose size is 60 × 40 in pixels. The goal of the navigation task is to let the robot move from any possible start position to the target position without colliding into obstacles. In this work, we limit the navigation task with fixed start and target positions, as shown in Figure 8a. The starting position (represented by the black cross) is in the lower left of the environment and the target (represented by the red star) is in the upper right of the environment. In Figure 8a, each PC-GWR node is labeled with a particular number, which corresponds to its one-hot representation during training (described below).

During learning, the robot is trained to solve the navigation task by only using its visual system. Related training settings are described as follows:

The robot’s states are represented by the place cell activities which encode locations in the environment and are calculated by inputting the image at the current position to the trained PC network. At the same time, the robot’s position is also represented by the best matching node in the PC-GWR. The PC-GWR provides a high-level representation of the environment and its nodes are the potential subgoals for the robot to choose from. The subgoal is represented by a one-hot vector whose length equals the number of nodes in the PC-GWR. Each element of the one-hot vector corresponds to a particular node in the PC-GWR.

The robot moves at a constant speed (0.2 pixels/s) and selects one of the possible actions that correspond to the eight compass directions as shown in Figure 3a.

The extrinsic and intrinsic rewards are defined as follows:

- During an episode, the agent receives an extrinsic reward of 2 when it reaches the target and −1 when a collision is detected, where both situations will immediately terminate the episode. To avoid unnecessary subgoal switches and encourage short trajectories to the goal, the agent receives an extrinsic reward of −0.05 for each state change on the high level.
- During the subgoal reaching, the agent receives an intrinsic reward of 0.2 when the subgoal is reached, or −1 when a collision is detected. If the moving step reaches the maximum number of steps (100 steps in our experiments), the episode terminates without a positive reward. This encourages the high-level policy to always select the nearby subgoals. In addition, for a faster subgoal reaching, the agent receives an intrinsic reward of −0.01 for each moving step.

During the HRL learning, for each episode, the robot starts with a random position and orientation in the simulator and ends either when the target is reached or when the robot collides with objects in the environment. The maximum moving steps in an episode are 1000 steps. The robot interacts with the simulator episodes by episodes and obtains a large number of experiences, which include both the state transitions in the space of place cell activities and the node transitions in the PC-GWR. Through learning from these experiences, the parameters of the HRL model are continuously updated until the optimal action policy to the target is obtained. The network architectures of the H_DQN and L_DQN are presented in Figure 8b. The input to the H_DQN is the ensemble activity of 30 place cells and the 14 outputs refer to the possible 14 subgoals. The input to the L_DQN is the ensemble activity of 30 place cells together with the one-hot subgoal representation and its eight outputs represent the Q-values with respect to the eight primitive actions. To train the network, we use the Adam optimizer and the learning rate $\theta_{H}$ and $\theta_{L}$ are set to be 0.0005 and 0.0001, respectively, both with a discount rate of 0.99. The subgoal/action selection policy is based on the ϵ-greedy with ϵ annealed from 1 to 0.1 over the duration of training. The sizes of the experience memories $D_{H}$ and $D_{L}$ are set to be 3000 and 8000, respectively, and mini-batches of 32 are used to select randomly retrieve experiences from these memories for learning and updating the neural network parameters. Additionally, we adopt the two-phase training procedure described in [42]. (1) In the first phase, the exploration parameter $\epsilon_{H}$ of the high-level policy is set to 1 and only the low-level policy is trained. Particularly, we limit the moving steps of the low-level policy to avoid challenging long-distance subgoal reachings. This results in a pre-trained low-level policy that is able to effectively solve a subset of subgoals that are within a certain range of the current position. (2) In the second phase, the high-level and low-level policies are jointly trained.

#### 5.4.2. Simulation Results and Comparision

In this part, we let the robot act according to the policies derived from the training in order to test the learned subgoal- and action-selection policies. For comparison, we also present the learning results of three baseline systems. For the same navigation task, the first baseline system uses a standard RL approach, D-DQN, which learns action policy using extrinsic rewards only. The second and third baseline systems are almost the same as the one described above except that they use topological maps of different size, which consists of six and 30 PC-GWR nodes, respectively. These baseline systems adopt the same training procedures and parameters described above, where the first baseline can be regarded as an ablation study.

**Figure 9:**
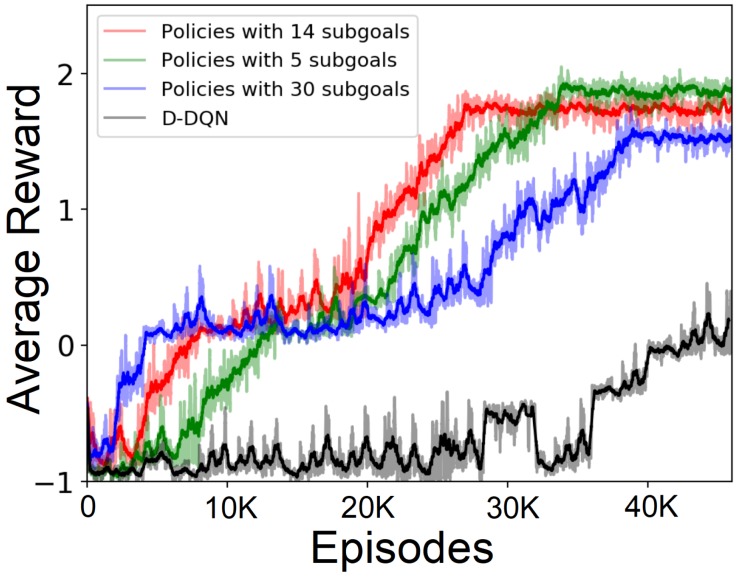
Learning curves of the robot under different learning paradigms, as functions of learning episodes.

Figure 9 shows the learning curves of different learning paradigms over the learning process. The curves demonstrate the total reward that the robot would receive under the policy learned at that point of the learning process and are all averaged over ten learning runs. We only show the learning curves up to the 44,000 episodes in order to compare the learning speed of different learning paradigms. As we can see, after about 40,000 episodes, the action policy learned by the standard RL still does not show good performance, while the policies learned using subgoals have all already converged. Note that the policy learned by the standard RL also converges to the same overall performance after about 75,000 learning episodes (not shown). This demonstrates the advantage of using the proposed HRL approach in solving navigation task in a large environment with sparse rewards.

Table 1 reports the evolution of the subgoals predicted by the H_DQN during the learning process. After about 30K learning episodes, we can see that the H_DQN is able to predict the correct subgoals for the robot to reach. This can be considered as the high-level planning on the PC-GWR.

Furthermore, we also compare the performance of learning using different numbers of subgoals (PC-GWR nodes) in the HRL paradigm. As shown in Figure 9, increasing the subgoal number from six to 14 increases the learning speed. However, increasing this number to 30 greatly decreases the learning speed. A PC-GWR with more nodes means a less sparse topological map which can represent the environment at a more fine-grained level. Although this could result in an easier subgoal reaching since the distance is relatively shorter, this makes the planning on the high level become much more complex. Meanwhile, it also means that the input space to the L_DQN is bigger, as shown in Figure 8b, which increases the training difficulty. This is the reason why keeping increasing the number of subgoals will not consistently increase the learning speed. Thus, in our learning approach, representing the environment with an appropriate abstraction level, i.e., using the PC-GWR with an appropriate size, is an important factor for efficient learning. Table 2 summarizes the final navigation performances of different learning paradigms.

**Figure 10:**
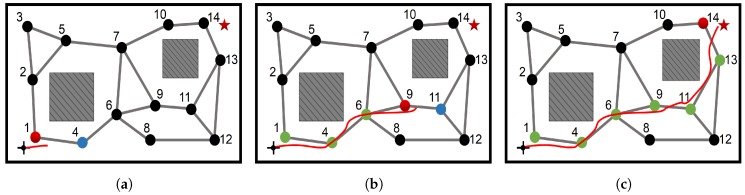
The navigation trajectory under the action policy after learning. (**a**) starting phase; (**b**) middle phase; and (**c**) ending phase. The robot’s current position is represented by the red node in the PC-GWR and its current subgoal is represented by the blue node. The red solid line represents the robot’s moving trajectory and the color of the passed PC-GWR nodes by the current time is changed into green.

Figure 10 presents the moving trajectory when the robot acts according to the learned policies during the navigation. The robot’s current position is represented by the red node in the PC-GWR and its current subgoal is represented by the blue node in the PC-GWR. For example, during the starting phase, the robot’s position is represented by node 1 in the PC-GWR and its current subgoal is node 4. During navigation, the robot moves towards the current subgoal step by step. Upon reaching the current subgoal (represented in the PC-GWR), the robot’s current state representation in the PC-GWR changes and a new subgoal is generated. This process repeats until the robot reaches the target location, during which nodes {4,6,9,11,13,14} are sequentially selected to be the subgoal. Notice that the robot always changes its subgoal before reaching the exact position of the subgoal. This is due to that each node in the PC-GWR represents an area rather than a single position. Thus, when moving towards a subgoal, the robot will consider itself already reaching the subgoal before reaching the exact position of the subgoal. However, the final navigation performance of our system is not affected by these localization errors. The program is running on a low-performance PC with an Intel Core i5-6200U CPU and the process time from receiving an image to generating an action command is about 0.75 s on average, which is sufficient for the real-time application.

## 6. Discussion

The proposed navigation system enables a robot to perform the goal-directed navigation based on learned place and HD cells. Compared with our previously proposed systems, the system present in this paper requires neither human efforts nor an accurate world model to perform planning. The navigational behaviors are learned automatically during interacting with the environment. Despite its success and efficiency, there are still several improvements can be made to the current system.

The SFA model is able to enable the self-organization of certain hippocampal cell types through learning from visual stimuli, in an attempt to reveal their formation mechanism from a computational perspective. However, it is a feed-forward model without any memory mechanisms, which means that it is certainly not sufficient to explain all the characteristics found in the hippocampus. For example, place cells have clearly demonstrated their essential roles in some memory-related tasks [68,69]. Also, our SFA model contains no recurrent connectivity [70] and path integration [71] mechanisms that are usually considered in theoretical hippocampal modelings. However, from a practical point of view, this simplicity also makes it outperform many other models in the way that it is convenient to model feature responses akin to those of hippocampal cell types in a real robotic context. And the type of learned cells solely depends on the relevant input statistics, which provides a big convenience for purposed learning. In this work, we aim to solve practical robotic tasks based on the learning representations from the SFA model, rather than elaborating the exact formation mechanisms of the hippocampal cells in animals’ brain.

Considering that the proposed system uses only a robot’s own onboard camera, it will be very easy to implement this system on a robot platform. Although the simulated robot uses a wide field of view (FOV) of about 320° to simulate a rat’s FOV [64], it has been claimed that small FOVs down to 60° could also produce the same place and HD cells [19]. We need to further test this considering the complexities of the real world such as changing lighting conditions and noisy sensory information. Besides, the simplicity of using only an RGB camera is also a big challenge to keep the accuracy and robustness of the system, which might lead to failures in the real world. To solve this, a promising solution is to resort to other sensors, like Lidar, depth camera, etc., to compensate for the limits of using only a camera. It will improve the completeness of our navigation system by integrating it with other sensory information.

In the current HRL paradigm, the low-level policy can be extended to work with a continuous action space, which is more natural and flexible in performing state transitions. Therefore, learning algorithms for continuous action spaces should be considered, such as Deep Deterministic Policy Gradient (DDPG) [27] and Asynchronous Advantage Actor-Critic (A3C) [72]. In addition, the current system can be extended to perform navigations in dynamic environments. Like many RL-based approaches, an open question to our current system is how to transfer the learned action policies from the simulation into the real world. Many works [73,74] solve this by training in extremely realistic, higher-fidelity simulated environments. However, our system works based on the SFA learning representations which are mainly related to the temporal feature among video sequences, rather the contextual information of a single image. This might have a loss requirement on the fidelity of the simulator. We will focus on this part in our future work.

## 7. Conclusions

In this paper, we have proposed a navigation system that enables a robot to navigate to the target position based on learning representations from its visual system only. It is a bio-inspired navigation system that involves modeling place and HD cells from the vision in an unsupervised fashion and performing navigation by implementing HRL on top of these cells’ activities. Specifically, we extend the traditional SFA model for purposeful learning of place and HD cells, by restricting the learning to separate movement phases. This enables to learn two distinct clusters of place cells and HD cells simultaneously through directly learning from visual inputs within just one exploration. Based on the learned different cell types, we build two topological maps using two GWR networks to learn separately from their activities, which enables the robot to perform localization and orientation detection. Furthermore, to accomplish the functional role of these hippocampal cells in spatial navigation, HRL is performed to learn action policies toward the target position, where the spatial state is represented by the ensemble activity of place cells and the hierarchy is provided by the topological map which is built in a self-organizing way. The HRL consists of a high-level policy working in the topological map to select subgoals and a low-level policy working in the space of the place cell activities to complete the selected subgoals. Experimental results have demonstrated the validity and efficiency of the proposed system in navigating a robot to the target position using just its visual system. Comparisons with baselines systems, e.g., RL, have shown the efficiency of the HRL in solving our navigation tasks. In future work, we will validate the proposed navigation system in the real world, which involves two challenging tasks: (1) modeling place and HD cells on a real robot using its camera; (2) transferring the learned policies in the simulation to the real world.

## Figures and Tables

**Table 1:** The subgoal predicted by the H_DQN at each state during the learning process.

PC-GWR Node	Subgoal Choice					
	Episode 0.2K	Episode 3K	Episode 10K	Episode 20K	Episode 30K	Episode 40K
1	4	2	2	4	4	**4**
2	12	3	1	5	5	5
3	8	2	1	2	5	5
4	8	1	8	1	**6**	**6**
5	6	7	2	3	7	7
6	7	4	8	7	**9**	**9**
7	3	10	9	5	10	10
8	11	5	9	6	12	12
9	5	6	7	11	**11**	**11**
10	14	7	14	14	14	14
11	2	12	12	13	**13**	**13**
12	9	11	8	8	13	13
13	4	14	14	11	**14**	**14**
14	3	12	14	14	**14**	**14**

**Table 2:** Performance of the learned policies in different learning paradigms.

Paradigm	Subgoal Number	Convergence Episodes	Reward	Avg. Trajectory Length
Paradigm 1	6	34.7k	1.85	450.3
Paradigm 2	14	27.6k	1.7	426.7
Paradigm 3	30	39.2k	1.4	510.8
D-DQN (44K)	0	–	−0.1	875.3
D-DQN (Converged)	0	75.0k	2.0	370.6
